# USP9X expression correlates with tumor progression and poor prognosis in esophageal squamous cell carcinoma

**DOI:** 10.1186/1746-1596-8-177

**Published:** 2013-10-23

**Authors:** Jing Peng, Qian Hu, Weiping Liu, Xiaoli He, Ling Cui, Xinlian Chen, Mei Yang, Hongqian Liu, Wei Wei, Shanling Liu, He Wang

**Affiliations:** 1 Laboratory of Genetics, West China Institute of Women and Children's Health, West China Second University Hospital, Sichuan University, Chengdu 610041, China; 2 Laboratory of Cell and Gene Therapy, West China Institute of Women and Children's Health, West China Second University Hospital, Sichuan University, Chengdu 610041, China; 3 Department of Obstetric and Gynecologic, West China Second University Hospital, Sichuan University, Chengdu 610041, China; 4 Key Laboratory of Obstetric and Gynecologic and Pediatric Diseases and Birth Defects of Ministry of Education, Chengdu 610041, China; 5 Department of Pathology, West China Hospital of Sichuan University, #37 Guoxue street, 610041 Chengdu, Sichuan, China; 6 Department of Obstetric and Gynecologic, Henan Provincial People's Hospital, Zhengzhou, Henan 450003 China; 7 Department of Gynecological Oncology, Second People's Hospital of Sichuan (Sichuan Cancer Hospital), Chengdu, Sichuan 610041, China

**Keywords:** Ubiquitin-specific protease 9x, Esophageal squamous cell cancer, Tumor progression, Survival

## Abstract

**Background:**

Ubiquitination is a reversible process of posttranslational protein modification through the action of the family of deubiquitylating enzymes which contain ubiquitin-specific protease 9x (USP9X). Recent evidence indicates that USP9X is involved in the progression of various human cancers. The aim was to detect the expression of USP9X in the progression from normal epithelium to invasive esophageal squamous cell cancer (ESCC) and evaluate the relevance of USP9X expression to the tumor progression and prognosis.

**Methods:**

In this study, USP9X immunohistochemical analysis was performed on tissues constructed from ESCC combined with either normal epithelium or adjacent precursor tissues of 102 patients. All analyses were performed by SPSS 13.0 software.

**Results:**

We observed that the level of high USP9X expression increased gradually in the transformation from normal epithelium (4.0%), to low grade intraepithelial neoplasia (10.5%), then to high grade intraepithelial neoplasia (28.6%), and finally to invasive ESCC (40.2%). The expression of USP9X was found to be significantly different between the normal mucosa and ESCC (P < 0.001), and between low grade intraepithelial neoplasia and high grade intraepithelial neoplasia (p = 0.012). However, no difference was observed between the high expression of USP9X in normal mucosa and low grade intraepithelial neoplasia (P = 0.369), nor between high grade intraepithelial neoplasia and ESCC (p = 0.115). Interestingly, the most intensive staining for USP9X was usually observed in the basal and lower spinous layers of the esophageal epithelium with precursor lesions which often resulted in the earliest malignant lesion. USP9X expression status was positively associated with both depth of invasion (p = 0.046) and lymph node metastasis (p = 0.032). Increased USP9X expression was significantly correlated to poorer survival rate in ESCC patients (p = 0.001). When adjusted by multivariate analysis, USP9X expression (HR 2.066, P = 0.005), together with TNM stage (HR 1.702, P = 0.042) was an independent predictor for overall survival.

**Conclusions:**

Up-regulation of USP9X plays an important role in formation and progression of precancerous lesions in ESCC and USP9X expression levels were significantly correlated with the survival of ESCC patients. Thus, USP9X could be considered as a potential biomarker and prognostic predictor for ESCC.

**Virtual slides:**

The virtual slides for this article can be found here: http://www.diagnosticpathology.diagnomx.eu/vs/1945302932102737

## Background

Esophageal squamous cell cancer (ESCC) is one of the most common lethal tumors in the world due to advanced disease, local relapse, distant metastasis, and resistance to adjuvant therapy [1-3]. Normal esophageal squamous epithelia undergo both genetic and histological changes during the evolution of ESCC, which involves a multistage process from noninvasive precursor lesions, initially containing low grade intraepithelial neoplasia, then containing high grade intraepithelial neoplasia, and finally towards invasive carcinoma [4]. Although certain events have been reported to occur during this process [5,6], the mechanisms regulating the malignancy and progression of ESCC remain under investigation [7,8].

Ubiquitination has been found to be a key regulatory mechanism in multiple biological processes and controls almost all aspects of protein function through the reversible posttranslational modification of cellular proteins by the action of ubiquitylating and deubiquitylating enzymes (DUBs) [5,9]. More attention has turned to the wide functional diversity of DUBs because they have a profound impact on the regulation of multiple biological processes including cell-cycle control, DNA repair, chromatin remodeling and several signaling pathways that are frequently altered in tumor development [10-12]. Ubiquitin-specific proteases (USPs), the largest group of DUBs, have fundamental roles in the ubiquitin system through their ability to specifically deconjugate ubiquitin from ubiquitylated substrates [13]. USP9X (ubiquitin-specific protease-9), one member of the USPs family, is widely expressed in all tissues with a large 2547-amino-acid-residue [14]. Overexpression of USP9X is reported in follicular lymphoma, diffuse large B-cell lymphoma and multiple myeloma [15]. In the same study, increased USP9X in multiple myeloma patients correlates with poor survival and the authors conclude that USP9X stabilizes MCL1, one member of pro-survival BCL2 family, and promotes tumor cell survival [15]. Afterwards, a partly selective DUB inhibitor WP1130 effectively downregulates anti-apoptotic and upregulates pro-apoptotic proteins by blocking the DUB activity including USP9X [5,16]. Moreover, WP1130 has also been found to promote Mcl-1 degradation and increases tumor cell sensitivity to chemotherapies in colon adenocarcinomas and lung cancers [17].

Nevertheless, to our knowledge, no direct evidence of USP9X in ESCC has been provided so far. The expression of USP9X and the exact role in the evolution of ESCC are far from understood. In the present study, we investigated USP9X expression and its potential clinical significance in normal esophageal epithelium, ESCC and its precursor lesions, trying to clarify the possible function of USP9X in the cancer malignancy, progression and prognosis.

## Materials and methods

### Tissue sample collection

ESCC combined with normal epithelium or adjacent precursor lesions from 102 patients were collected in the Pathology Department of West China Hospital, Sichuan University from Jan, 2001 to Jan, 2003. Patients receiving chemotherapy or radiation therapy before esophagectomy were excluded. Among the 102 patients, only 25 cases were ESCC combined with normal mucosa. These 25 had no precursor lesions. Of the remaining 77 cases, there were 20 cases of ESCC combined with low grade intraepithelial neoplasia, 17 cases were ESCC combined with high grade intraepithelial neoplasia, and 18 cases were ESCC combined with both precursor lesions. The remaining 22 cases were ESCC with no other complications. All tumors had been confirmed by postoperative histopathologic assessment. Staging in esophageal cancer was principally based on the International Union Against Cancer Classification of 2010 [18] while evaluation of tumour differentiation was based on histological criteria of the guidelines of the World Health Organization Pathological Classification of Tumors. Overall survival was calculated from the date of surgery to the date of death or the last follow-up. All patients were followed until death or the end of the follow-up period (March, 2012).

The study was approved by the ethics committee of West China Second Hospital of Sichuan University and informed consent was obtained from all patients undergoing surgery.

### Immunohistochemistry

The slides were first baked at 37°C overnight and deparaffinized in xylene, then rehydrated in graded ethanol. High-temperature antigen retrieval was performed in a 10 mmol/L boiling sodium citrate buffer at pH 6.0 for 15 min. The slides were cooled to room temperature and then immersed in 3% hydrogen peroxide for 30 min to block endogenous peroxidase activity, and incubated in 10% normal goat serum for 30 min to reduce nonspecific binding. Excess blocking solution was discarded, the sections were incubated with monoclonal mouse anti-human USP9X antibody (diluted 1:150, NBP2-03824, NOVUS Biologicals) at 4°C overnight. The sections were first washed with PBS and then incubated with biotinylated secondar (SP-9002, Zhongshan Golden Bridge Inc., China) for 60 min at room temperature. Slides were then treated with streptavidin peroxidase for 60 min at room temperature, followed by incubation with DAB (3, 3’-diaminobenzidine solution). Cells with brown staining in the cytoplasm were considered positive. The slides were then counter-stained with hematoxylin and mounted with neutral balsam. Additionally, sections incubated with normal serum blocking. Omission of the primary antibodies were considered as blank controls, confirming any nonspecific staining.

### Evaluation of USP9X protein expression

For evaluation of USP9X protein expression, a reproducible semiquantitative method that takes both staining intensity (0, negative; 1, weak; 2, moderate and 3, strong) and percentage of positive cancer cells (0, none; 1, <10%; 2, 10–50%; 3, 51 –80%; 4, > 80%) into account was adopted. The final score was calculated by adding scores for percentage and intensity of positive cells. Scores of 0 ~ 3 were defined as “negative expression” (-), scores of 4 ~ 5 as “weakly positive expression” (+), and scores of 6 ~ 7 as “strongly positive expression” (++) [7]. Additionally, overall scores were divided into two groups: low expression (0-5) and high expression (6 –7) in ESCC samples.

### Statistical analysis

The association of clinicopathologic characteristics with USP9X expression status was analyzed by the Pearson’s χ2 test or Fisher’s exact test for categorical variables. The Kaplan–Meier method and the log-rank test were performed to assess the cumulative survival rate. Univariate and multivariate Cox proportional hazard models were used to estimate the relationship between USP9X expression and clinical characteristics to overall survival. Variables for multivariate analysis were selected by means of a stepwise forward selection method. All analyses were performed by SPSS 13.0 software (SPSS Inc., Chicago, USA). *P* values of less than 0.05 were considered statistically significant.

## Results

### USP9X expression in normal esophageal squamous epithelia, intraepithelial neoplasia, and ESCC detected by immunohistochemistry

**Figure 1:**
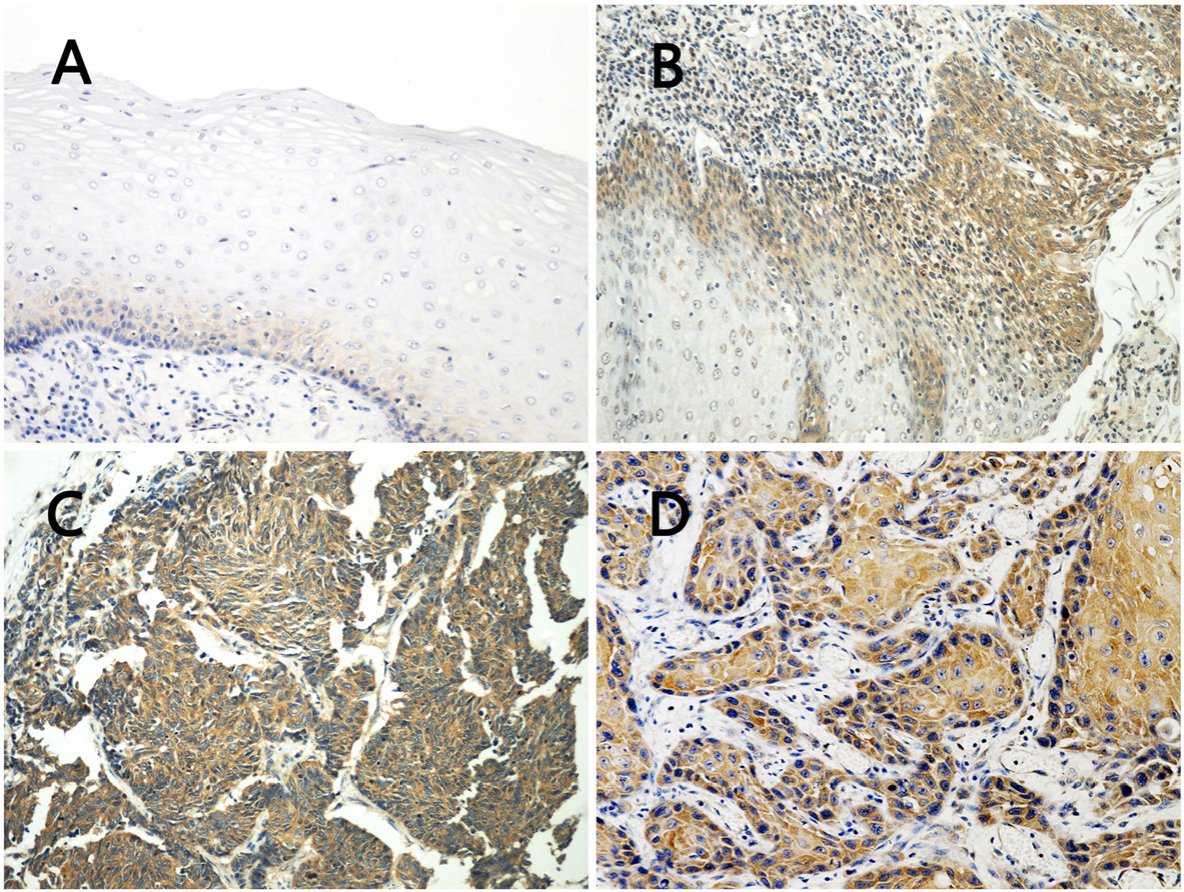
**Immunohistochemical staining of USP9X expression in the progression from normal epithelium to ESCC.** Paraffin-embedded tissue sections were stained using an immunoperoxidase method, as described in Materials and methods. Representative images (200×) are shown: **A** In normal esophageal epithelium, immunostaining for weak USP9X signal was found only in the basal layer. **B** In low grade intraepithelial neoplasia (left side), positive staining was observed in most of the heterogeneous cells from the basal layer to the granular layer of epithelium. The USP9X expression increased gradually in the transformation from low grade intraepithelial neoplasia to high grade intraepithelial neoplasia as carcinoma *in situ* in which the full-thickness epithelium showed diffuse immunoreactivity for USP9X (right side). **C, D** In ESCC, intense immunostaining for USP9X was presented in the cytoplasm of most of the cancer cells.

As shown in Figure 1, positive immunostaining for USP9X could be observed in a cytoplasmic pattern. In normal epithelium, weak positive signals were seen only in the basal layer and some of the lower spinous layer in the epithelium, whereas in precursor lesions positive staining was observed in most of the heterogeneous cells of the epithelium (Figure 1A,B). We also noticed that the USP9X expression increased gradually in the transformation from low grade intraepithelial neoplasia to high grade intraepithelial neoplasia as carcinoma *in situ* in which the full-thickness epithelium showed intensive immunostaining for USP9X (Figure 1B). Moreover, most of ESCC showed diffusely strong positive immunostaining of USP9X (Figure 1C,D).

USP9X expression in normal esophageal squamous epithelium, different precursor lesions and ESCC was summarized in Table 1. As much as 96.0% of normal tissue samples were detected with USP9X expression at a negative or low level, whereas in ESCC tissues high USP9X expression was 40.2%. The expression of USP9X was found significantly different between ESCC and the normal mucosa (P < 0.001). However, both between normal mucosa and low grade intraepithelial neoplasia (P = 0.369), and between high grade intraepithelial neoplasia and ESCC (P = 0.115), no significance was detected in the high expression of USP9X. Nevertheless, there was a significance in USP9X expression between low grade intraepithelial neoplasia and high grade intraepithelial neoplasia (P = 0.012). Moreover, a gradual increase of positive rate in high USP9X protein staining from normal (4.0%) to precancerous (low grade intraepithelial neoplasia: 10.5%, high grade intraepithelial neoplasia: 28.6%) and carcinoma tissues (40.2%) was clearly detected, demonstrating that USP9X protein expression might indicate the progress of ESCC.

**Table 1:** Summary of immunohistochemical expression of USP9X in different lesions

**Group**	**USP9X staining**			**Value**			
	**- (%)**	**+ (%)**	**+ + (%)**	**P**^**a**^	**P**^**b**^	**P**^**c**^	**P**^**d**^
Normal	18 (72.0)	6 (24.0)	1 (4.0)				
Low grade intraepithelial neoplasia	21 (55.3)	13 (34.2)	4 (10.5)				
High grade intraepithelial neoplasia	8 (22.9)	17 (48.6)	10 (28.6)				
Cancer	10 (9.8)	51 (50.0)	41 (40.2)	0.37	0.01	0.12	<0.001

^a^Esophageal normal epithelia VS. low grade intraepithelial neoplasia.

^b^Low grade intraepithelial neoplasia VS. high grade intraepithelial neoplasia.

^c^High grade intraepithelial neoplasia VS. ESCC.

^d^Esophageal normal epithelia VS. ESCC.

### Correlation between USP9X expression and ESCC Clinicopathological parameters

As shown in Table 2, ESCC samples with high-expression of USP9X had significantly higher frequencies of T3–T4 cases compared with the low-expression group (51.1% vs. 31.6%, respectively; P = 0.046) and high expression of USP9X was more prevalent in node-positive than in node-negative cases (51.0% vs. 30.2%, respectively; P = 0.032). TNM stage did not reach any statistical significance with USP9X expression; however, it displayed a clear trend (P = 0.112). We also observed a trend between USP9X expression and histological grade, although this was not statistically significant (P = 0.123).

**Table 2:** ESCC patient characteristics and USP9X expression

**Characteristic**	**Total (n = 102)**	**No. of USP9X positive expression cases (%)**	**P value**
Age (years)			0.961
≤59	60	24 (40.0)	
≥60	42	17 (40.5)	
Gender			0.928
Male	85	34 (40.0)	
Female	17	7 (41.2)	
Location			0.763
Upper	6	2 (33.3)	
Middle	75	29 (38.7)	
Lower	21	10 (47.6)	
Tumor length			0.717
≤5 cm	60	25 (41.7)	
>5 cm	42	16 (38.1)	
Histological grades			0.123
G1^a^	22	13 (59.1)	
G2^a^	58	20 (34.5)	
G3^a^	22	8 (36.4)	
Depth of invasion			0.046
T1-T2	57	18 (31.6)	
T3-T4	45	23 (51.1)	
Lymph node metastasis			0.032
Positive	49	25 (51.0)	
Negative	53	16 (30.2)	
TNM stage			0.112
I-II	57	19 (33.3)	
III-IV	45	22 (48.9)	

^a^G3, well-differentiated carcinoma; G2, moderately-differentiated carcinoma; G1, poorly-differentiated carcinoma.

### Survival analysis and prognostic significance of USP9X expression in ESCC

**Figure 2:**
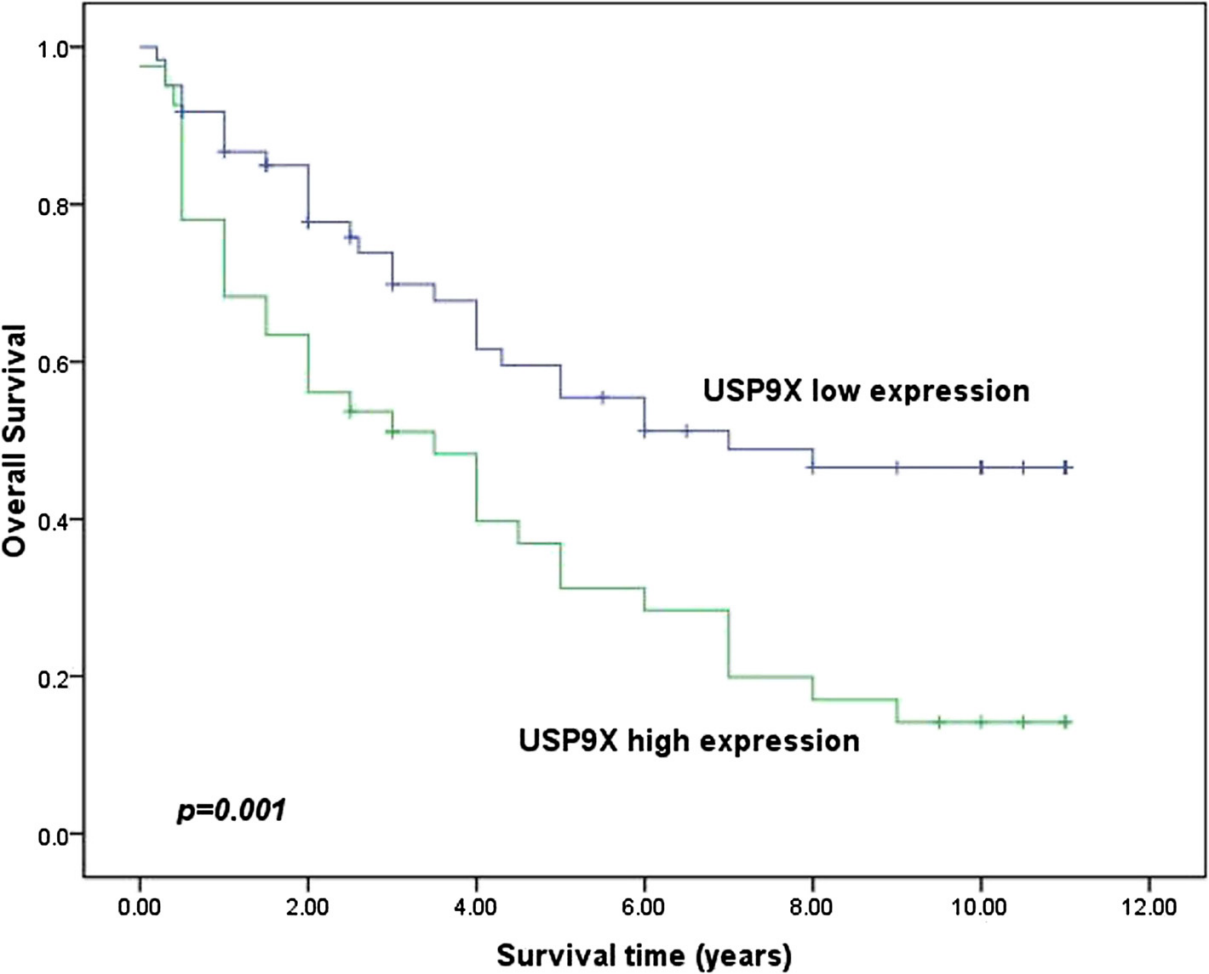
**Relationship between USP9X expression status and cumulative survival in ESCC (n = 102).** The 5-year survival rate of patients with high USP9X expression was 31.2%, compared to 55.4% for patients with low USP9X expression (P = 0.001).

Survival analysis was conducted through Kaplan–Meier curves for general survival. The overall survival rate of patients with high USP9X expression was significantly lower than that of patients with low USP9X expression. The 5-year overall survival rate of the high-expression group was 31.2%, compared to 55.4% in the low-expression group. Moreover, the 10-year survival rate of patients with high USP9X expression was 14.2%, compared to 46.5% in patients with low USP9X expression (P = 0.001; Figure 2).

Further, in a univariate Cox regression analysis in ESCC patients, the poor overall survival correlated with depth of invasion (HR 1.740, P = 0.033), regional lymph node metastasis (HR 1.910, P = 0.015), TNM stage (HR 1.842, P = 0.018) and USP9X expression (HR 2.192, P = 0.002). Moving ahead with the multivariate analysis, we noted that both TNM stage (HR 1.702, P = 0.042) and USP9X expression (HR 2.066, P = 0.005) remained significantly associated with overall survival (Table 3).

**Table 3:** Statistical analysis of cancer-specific survival of ESCC patients (n = 102)

**Factor**	**Univariate analysis**		**Multivariate analysis**	
	**HR (95% CI)**	**P value**	**HR (95% CI)**	**P value**
Age (≤59 vs. ≥60)	0.963 (0.573-1.617)	0.89		
Gender (male vs. female)	0.982 (0.498-1.936)	0.96		
Tumor length (≤5 cm vs. >5 cm)	1.002 (0.603-1.665)	1		
Histological grades (G3 vs. G2 vs. G1)	1.151 (0.801-1.654)	0.45		
Depth of invasion (T1-T2 vs. T3-T4)	1.740 (1.047-2.892)	0.03	—	0.36
Lymph node metastasis (negative vs. positive)	1.910 (1.137-3.209)	0.02	—	0.43
TNM stage (I-II vs. III-IV)	1.842 (1.109-3.062)	0.02	1.702 (1.020-2.838)	0.04
USP9X expression (low vs. high)	2.192 (1.322-3.634)	0	2.066 (1.242-3.437)	0.01

HR hazard ratio, CI confidence interval.

These findings showed that high expression of USP9X is associated with shorter survival in ESCC patients and indicated as an independent prognostic factor.

## Discussion

Esophageal squamous cell cancer is one of the most aggressive and deadly tumors in solid oncology. Despite major advances in the therapeutic approach to this disease, the crude mortality rate of esophageal cancer remained with a 5-year survival rate of 10% to 20% [19]. One of the reasons for its poor prognosis is that ESCC is difficult to diagnose at an early stage [20]. Therefore, it would be of great clinical benefit if the precursor lesions of ESCC could be detected early through potential biomarkers to promote the survival [21]. In clinical pathology, the precursor lesions of ESCC are thought to consist of various morphological stages: mild dysplasia, moderate dysplasia, severe dysplasia and carcinoma *in situ*. The mild dysplasia and moderate dysplasia are also called low grade intraepithelial neoplasia, while severe dysplasia and carcinoma *in situ* are defined as high grade intraepithelial neoplasia [7,22]. We speculate that some biological events that account for the malignancy and development of ESCC, and some molecules could be identified as prognostic biomarkers in precursor lesions.

USP9X is excessive in tumor tissues such as follicular lymphoma [15], colon adenocarcinomas and lung cancers [17] compared to the normal human tissues and has an impact on tumor progression. In the present study, we demonstrated the up-regulation of USP9X during the process of initiation and progression of ESCC for the first time. We observed that USP9X expression was significantly higher in ESCC (90.2%) than that in normal epithelium (28%). Furthermore, level of high USP9X expression increased gradually in the transformation from normal epithelium (4.0%), low grade intraepithelial neoplasia (10.5%), high grade intraepithelial neoplasia (28.6%), to invasive ESCC (40.2%). Although there was no difference between the high expression of USP9X in normal mucosa and low grade intraepithelial neoplasia (P = 0.369), nor between high grade intraepithelial neoplasia and ESCC (p = 0.115), significance was detected in USP9X expression between low grade intraepithelial neoplasia and high grade intraepithelial neoplasia (p = 0.012). Thus, we supposed USP9X correlated with the progression of ESCC and up regulation of USP9X might be a late event in the multistep pathogenesis of ESCC, because 55.3% of low grade intraepithelial neoplasia were not detected with USP9X expression, whereas 77.2% of high grade intraepithelial neoplasia had positive expression of USP9X (P = 0.012). The abnormal regulation and control of cell cycle in the basal layer cells of the epithelium often resulted in the earliest malignant lesion of the esophagus [23,24]. Interestingly, the most intensive staining for USP9X was usually observed in the basal and lower spinous layers of the esophageal epithelium with precursor lesions in our study. These probably indicated that up-regulation of USP9X plays an important role in formation and progress of precancerous lesions in ESCC, suggesting USP9X could be a potential biomarker for ESCC.

As is well known, the prognosis and choice of therapy for ESCC patients are determined primarily by the stage of disease [25]. Moreover, lymph-node metastasis has been reported to be an important negative prognostic indicator of ESCC and was often related to the depth of invasion [26,27]. In this study, we observed that USP9X expression status was well-associated with depth of invasion (p = 0.046) and lymph node metastasis (p = 0.032). Nevertheless, no statistical significance was found between USP9X expression and histological grade (p = 0.123) or TNM stage (p = 0.112) in ESCC, although it displayed a clear trend. Maybe the uneven distribution of patients in different histological grades and TNM stages biased the results. We further evaluated the prognostic value of USP9X in ESCC. The results showed that increased USP9X expression was significantly correlated to a lower survival rate in patients after radical surgery (P = 0.001). Importantly, TNM stage (HR 1.702, *P* = 0.042) and USP9X expression (HR 2.066, P = 0.005) were revealed as independent predictors of prognosis according to multivariate Cox regression analysis. Thus, USP9X could be considered as a potential diagnosis and prognostic predictor for ESCC.

Recent studies have addressed the possible relation between USP9X expression and clinicopathologic factors in human tumors. Interrogation of public expression databases [28,29] has shown that increased USP9X mRNA in tumors could significantly anticipate poor outcome for multiple myeloma patients. MCL1, one member of pro-survival BCL2 family, is rapidly turned over through the action of ubiquitin ligases [30,31]. Martin Schwickart et al. then indicated that interaction of USP9X and MCL1 is of prognostic relevance for several human malignancies including multiple myeloma [15]. They used USP9X knockdown in combination with ABT-737, a small molecule antagonist of the pro-survival proteins not including MCL1 to test increased apoptosis in tumor cells. They found USP9X knockdown alone caused a modest decrease in tumour growth but specifically stabilized MCL1 by removing its degradative Lys 48-linked polyubiquitin chains to promoting cell survival. However, another observation found that low USP9X protein and messenger RNA expression in pancreatic ductal adenocarcinoma (PDA) were inversely associated with poor survival after surgery [32]. What is more, obvious changes in Mcl1 protein levels could not be detected upon Usp9x loss in PDA. Maybe these opposing findings could be explained by the tissue-specificity of USP9X in different tumors. As the malignant development of different cell types may be somewhat different, the carcinogenesis of USP9X in different tissues also had its own characteristics. In this study, our results provided the first evidence that USP9X expression was associated with the progression and poor outcome in ESCC. However, further studies are necessary to precisely identify the molecular mechanisms.

## Conclusion

The present study offered clinical evidence for the first time that USP9X expression is well correlated to ESCC progression, aggressive behaviors and poor prognosis. Therefore, we envision that USP9X may be a novel tumor marker, a prospective prognostic indicator and a potential therapeutic target for ESCC.

## Competing interests

All the authors declare that there is no competing interests.

## Authors’ contributions

JP, SLL, HW and WPL designed the study and wrote the manuscript. QH, XLH and LC collected the patients’ clinical information and obtained the follow-up data. JP and XLC analyzed and interpreted the data. WPL, MY, HQL and WW revised the intellectual content. All authors have read and approved the final manuscript.
